# A higher dose of enzyme therapy in patients with classic infantile Pompe disease seems to improve ventilator-free survival and motor function

**DOI:** 10.1186/1471-2474-14-S2-P19

**Published:** 2013-05-29

**Authors:** C van Gelder, I Plug, M Kroos, A Reuser, A van der Ploeg

**Affiliations:** 1 Department of Pediatrics, Center for Lysosomal and Metabolic Diseases, Erasmus MC University Medical Center, Rotterdam, The Netherlands; 2 Department of Clinical Genetics, Center for Lysosomal and Metabolic Diseases, Erasmus MC University Medical Center, Rotterdam, The Netherlands

## Introduction

Enzyme replacement therapy (ERT) with Myozyme® has significantly improved the prospect for patients with classic infantile Pompe disease. Yet, about 50% of patients still do not survive ventilator-free beyond 2.5 years. In the present study we compared the safety and efficacy of treatment with 40 mg/kg/week to that of 20 mg/kg/ every other week (eow) in 10 infantile patients to determine if a higher/more frequent dose would improve outcomes. All patients were treated for at least one year and received the same dose throughout the study.

## Results

Our outcome parameters included survival, ventilator free survival, left ventricular mass index, motor outcome, and infusion associated reactions. A total of six patients received 20 mg/kg/eow, and four patients received 40 mg/kg/week. The median treatment duration was 3.5 years and 1.6 years, respectively. The median age at the start of therapy was 1.5 months for the 20 mg/kg group and 3.1 months for the 40 mg/kg group. During the treatment period, 3 of the 10 patients became respiratory insufficient. They all belonged to the 20 mg/kg group, and two of the three were CRIM negative. Four of six patients in the 20 mg/kg group learned to walk, but two later lost this ability after becoming ventilator dependent. In contrast, all patients from the 40 mg/kg dose group learned to walk and maintained the ability to walk, even though their baseline motor functioning was generally worse. The decrease in left ventricular mass index and the number of infusion associated reactions was comparable in both groups.

## Conclusion

The preliminary data of our study show that treatment with Myozyme at a higher dose of 40 mg/kg/week is generally well tolerated and leads to improved ventilator-free survival and motor outcomes than treatment with 20 mg/kg/eow.

